# Supplementary material for: Layer-polarized ferromagnetism in rhombohedral multilayer graphene
Source: Nat Commun. 2024 Mar 22;15:2597. doi: 10.1038/s41467-024-46913-5 (PMC10960043; doi:10.1038/s41467-024-46913-5)
Supplement: Supplementary file 1 — Supplementary Information [file 41467_2024_46913_MOESM1_ESM.pdf]

Supplementary Information for

Layer-polarized ferromagnetism in rhombohedral multilayer graphene

Wenqiang Zhou[1,2#], Jing Ding[1,2#], Jiannan Hua[1,2#], Le Zhang[1,2], Kenji Watanabe[3], Takashi Taniguchi[4], Wei Zhu[1,2*], Shuigang Xu[1,2*]

[1] *Key Laboratory for Quantum Materials of Zhejiang Province, Department of Physics, School of Science, Westlake University, 18 Shilongshan Road, Hangzhou 310024, Zhejiang Province, China*

[2] *Institute of Natural Sciences, Westlake Institute for Advanced Study, 18 Shilongshan Road, Hangzhou 310024, Zhejiang Province, China*

[3] *Research Center for Electronic and Optical Materials, National Institute for Materials Science, 1-1 Namiki, Tsukuba 305-0044, Japan*

[4] *Research Center for Materials Nanoarchitectonics, National Institute for Materials Science, 1-1 Namiki, Tsukuba 305-0044, Japan*

[#]These authors contributed equally to this work.
[*]Correspondence to: zhuwei@westlake.edu.cn, xushuigang@westlake.edu.cn

Supplementary Note 1: Layer number determination

The layer number of graphene was determined through reflection contrast spectroscopy[1]. We mechanically exfoliated multilayer graphene from bulk crystals (NGS Naturgraphit) onto standard SiO_2/Si substrates, with the oxide layer thickness of 285 nm. Supplementary Figure 1a shows an optical image of a 7L graphene flake with multiple steps at its edge. The optical contrast of various layers relative to the adjacent substrate near the edge is shown in Supplementary Figure 1b. This optical contrast follows the Beer-Lambert law, exhibiting a linear increase relative to the layer number, as shown in Supplementary Figure 1c. We find this thickness-dependent contrast is consistent across different flakes, allowing it to be utilized for identifying multilayer graphene even in the absence of stepped edges.

Supplementary Note 2: Raman spectroscopy

To elucidate the impact of moiré potential on the surface and bulk states of 7L graphene, we conducted a comparative analysis of Raman spectra. We examined both aligned and non-aligned 7L graphene, encompassing ABA and ABC stacking domains.

It's well known that in the case of monolayer and bilayer graphene, the moiré superlattice formed between graphene and h-BN can induce a periodic strain distribution, resulting in a noticeable broadening of the 2D peak[2,3]. For instance, in monolayer graphene, when singly aligned with h-BN, the full width at half-maximum (FWHM) of the 2D peak exhibits a value of approximately 20 cm^{-1} larger than non-aligned counterpart. This broadening becomes even more pronounced, reaching an increase of about 40 cm^{-1} for doubly aligned monolayer graphene in comparison to the non-aligned one[4].

However, in the case of ABC graphite, we observe no distinguished broadening of the 2D peak between aligned and non-aligned configurations. As shown in Supplementary Figure 3c, the FWHM exhibits similar value of ~ 70 cm^{-1}. This feature is consistent with a recent report on ABA-stacked graphite[5], which also demonstrates a very short penetration depth for moiré reconstruction.

Supplementary Note 3: Twist angle determination

To accurately determine θ, we employed Brown–Zak oscillations occurring at a moiré superlattice under high magnetic fields (B)[6,7]. In Fig. 2c, we observed remarkable quantum oscillations with periodicities independent of n with a fixed $D = 0$ V nm^{-1}. Further analysis reveals that the minima in R_{xx} exhibit two sets of oscillatory behavior periodic in $1/B$, characteristic of Brown–Zak oscillations[8]. In systems with superlattices under a magnetic field, the electronic spectra can develop into fractal spectra known as Hofstadter butterflies, resulting in a series of minima R_{xx} at $B = \phi_0/qA$, where q is an integer, ϕ_0 magnetic flux quantum, and $A = \sqrt{3}\lambda^2/2$ the unit-cell area of the superlattice. The low resistance observed in Brown–Zak oscillations stems from the repetitive formation of magnetic Bloch states at magnetic field following the sequence of $\frac{\phi}{\phi_0} = 1/q$, in which electrons recover delocalized wave functions and propagate along open trajectories instead of cyclotron trajectories. The two distinct sets of Brown–Zak oscillations in Fig. 2c, indicate that both the top and bottom h-BN are aligned with graphene in device D2[9,10]. By individually fitting these oscillations, we extracted two moiré wavelengths as $\lambda_1 = 11.1$ nm and $\lambda_2 = 11.0$ nm, corresponding to two twist angles $\theta_1 = 0.88°$ and $\theta_2 = 0.90°$ at the bottom and top interfaces.

The θ can also be calculated from resistance peaks at n_1 and n_2 corresponding to $\nu = 1$ and $\nu = 2$. Given the four-fold degeneracy (two for spin and two for valley) in graphene, four electrons per moiré cell are required for full filling of a moiré miniband ($\nu = 4$). The corresponding n at $\nu = 4$ is $n_s = 4n_1 = 2n_2 \approx 8\theta^2/\sqrt{3}a_G^2$. The twist angles calculated from this method are 0.90° and 0.94°, approximately consistent with those extracted from Brown–Zak oscillations.

Supplementary Note 4: LLs at high field

In ABA-stacked graphite thin film, at high B, when the system enters into the ultra-quantum regime, only the two lowest Landau bands (0 and 1) are across the Fermi energy. Within this regime, electrons form a set of standing waves along the c-axis, penetrating across the entire bulk owing to the one-dimensional Landau bands because of a finite thickness of graphite films. These states are thus subject to the influence of both top and bottom gates, resulting in the diagonal features at center of $R_{xx}(n_t, n_b)$ map. Meanwhile, the horizontal and vertical features at the edge of $R_{xx}(n_t, n_b)$ map are attributed to quantized states at the graphite surfaces, coexisting with the screening bulk states[5,11]. These features occur at both standard quantum Hall effect and Hofstadter's butterfly gaps, evidenced by recent observations that the moiré surface potential affects the entire bulk of graphite in the ulta-quantum regime[5,12].

In rhombohedral multilayer graphene, the situation is quite different. At moderate B, The Landau diagrams plotted in $R_{xx}(n_t, n_b)$ (as shown in Fig. 3) exhibit a series of horizontal and vertical

features at small D, whereas these features transform into diagonal patterns at high D. These characteristics reveal the decoupled surface states inherent to rhombohedral 7L graphene. At low temperature, the electronic transport properties of rhombohedral multilayer graphene are dominated by its surface states, with the conductivity through the 3D bulk band being effectively suppressed[13]. Specifically, at small D, there is a finite band overlap between the conduction and valence bands, leading to the presence of two metallic surface states (the correlation gap at $n = 0,\ D = 0$ is smeared out by the strong magnetic field). In this scenario, the gate-voltage-induced surface charge accumulation on one of the two surfaces exerts a strong screening effect, diminishing its gating effect on the other surface. Consequently, the two surface states become electronically decoupled. However, at high D, the inversion symmetry breaking overcomes the band overlap, effectively polarizing the surface states. When fixing D and tuning n, only one of the two surfaces become conductive, and this conductivity can be effectively tuned by both gates. As a result, we observe the emergence of diagonal LLs at high D, as shown in Fig. 3.

Supplementary Note 5: $N\pi$ Berry phase

In rhombohedral N-layer graphene, low-energy electrons are localized on the top and bottom layers. A simplified two-band model approximately describes the low-energy bands with a dispersion relation of $E \sim \pm p^N$, exhibiting a large DOS at $n \to 0$ and a $\pm N\pi$ Berry phase around K/K' valley. The valley and layer are strongly coupled such that K (K') valley associated with $N\pi$ $(-N\pi)$ Berry phase is localized on the top (bottom) layer. Under the influence of B, the low-energy bands in rhombohedral graphene develop into a series of LLs. The zeroth LL has N-fold orbital degeneracy, two-fold valley degeneracy, and two-fold spin degeneracy. This LL evolves with B as $E^{\pm} \propto \pm B^{N/2}$. It's worth noting that the valley degeneracy in the zeroth LL is equivalent to layer degeneracy. In the case of rhombohedral 7L graphene, the zeroth LL has a total degeneracy of 28, corresponding to the filling factor from -14 to +14. The application of an external D breaks the inversion symmetry and lifts the valley degeneracy. Consequently, the zeroth LL is split as -7 to +7. This layer-number dependent orbital degeneracy serves as an indicator of the layer number in rhombohedral multilayer graphene devices. Additionally, it also provides evidence for the existence of $N\pi$ Berry phase in rhombohedral N-layer graphene.

Experimentally, high-order hopping terms distort the simple power-law low-energy bands in terms of trigonal warping and electron-hole asymmetry, which break the orbital degeneracy of the zeroth LL and induce a series of LLs crossings at the hole side.

In Supplementary Figure 6, we present the observed manifestation of a 7π Berry phase in rhombohedral 7L graphene, in the absence of a moiré superlattice. Specifically, in the absence of external displacement filed ($D = 0$ V nm^{-1}), pronounced $\nu = -14$, corresponding to the gap between zeroth LL and first LL, is marked. Within the zeroth LL, we observed both orbital splitting and spin splitting at high B. Within the region of n between -3.5×10^{12} cm^{-2} and -5.5×10^{12} cm^{-2}, the zeroth LL overlaps with the valence-band LLs, resulting in intricate quantum oscillations. At sufficiently high hole density and large B, the simple four-fold degeneracy characteristic of valence-band LLs is recovered. The introduction of a finite D results in the lifting of valley degeneracy due to the breaking of inversion symmetry. As a result, pronounced $\nu = -7$ emerges, confirming the presence of a 7π Berry phase in rhombohedral 7L graphene.

In general, the $N\pi$ Berry phase related quantum Hall states are inherent to rhombohedral multilayer graphene. We also observed similar 6π Berry phase induced $\nu = -12$ states in rhombohedral hexalayer (6L) graphene (Supplementary Figure 8b), resembling those reported in rhombohedral 9L or thicker graphene previously reported[13].

Supplementary Note 6: Spontaneous symmetry breaking in non-aligned rhombohedral multilayer graphene

In intrinsic rhombohedral multilayer graphene without moiré, the surface flat band near Fermi surface favors interaction-driven symmetry breaking. In the non-interacting regime, this band has a four-fold degeneracy due to the presence of spin and valley symmetries. This can be revealed from the period of Shubnikov–de Haas (SdH) oscillations at relatively low *B*, calculated as $\Delta v = \frac{\Delta n h}{eB}$. As shown in Supplementary Figure 7b, at high *n* we observed $\Delta v = 4$, consistent with expectations from the single-particle picture. The application of *D* can further flatten the surface band, dramatically increasing DOS near vHSs. When the Stoner criterion $UD_F > 1$ is satisfied at specific *n* and *D*, spontaneous spin-valley flavor polarization occurs. In this situation, the initial four-fold degeneracy is reduced to two-fold in the case of a half-metal state (spin or valley polarized) or fully lifted in the case of a quarter-metal state (spin and valley polarized). This degeneracy lifting can be observed both in the n-D mapping at a fixed B (Supplementary Figure 7b) and in quantum oscillations as a function of B (Supplementary Figure 7c). When in a half-metal state, we observed AHE, providing evidence of valley polarization occurring as B approaches zero. This valley polarization gives rise to a nonzero Berry curvature, leading to an intrinsic AHE. It's worth noting that the valley-polarized half-metal state observed in this study is distinct from those in thinner rhombohedral graphene systems[14-16], where AHE was only observed in quarter-metal region. Our observations in 7L graphene offer valuable insights into the evolution of Stoner instability with increasing layer number. Notably, the pronounced screening effect between two surface states observed in the low D region (see Fig. 3 and Supplementary Figure 7b), absent in previously reported thinner rhombohedral graphene, strongly indicates that the two surface states 7L graphene are decoupled under magnetic field.

Though the half-metal state with AHE emerges in the intrinsic rhombohedral 7L graphene without a moiré superlattice, it's noted that this state appeared at a very narrow region. The introduction of moiré superlattice in rhombohedral 7L graphene can further flatten the surface band and favor the spontaneous symmetry breaking. This, in turn, facilitates the emergence of layer-polarized ferromagnetism across a significantly large region, as shown in Fig. 4.

The spontaneous symmetry breaking states can occurs beyond 7L. In Supplementary Figure 8c, we show the half-metal and quarter-metal states in intrinsic rhombohedral 6L graphene.

Supplementary Note 7: Band structure calculation

Rhombohedral 7L graphene with moiré superlattice has Hamiltonian

$$H_{tot} = H_7 + V_{mo},$$

where H_7 is the effective tight-binding Hamiltonian of the intrinsic rhombohedral 7L graphene,

and the effective intralayer moiré potential V_{mo} is only applied to the graphene layer contacting with h-BN layer[17].

Using the Slonczewski-Weiss-McClure (SWMC) tight-binding lattice model, the Hamiltonian can be written as

$$H_7 = \begin{pmatrix} D_1 & V & W & 0 & 0 \\ V^\dagger & D_2 & V & \ddots & 0 \\ W^\dagger & V^\dagger & \ddots & \ddots & W \\ 0 & \ddots & \ddots & D_6 & V \\ 0 & 0 & W^\dagger & V^\dagger & D_7 \end{pmatrix} \text{……………Supplementary Equation 1}$$

where the 2×2 blocks are

$$D_i = \begin{pmatrix} u_{Ai} + \delta_i & v_0\pi^\dagger \\ v_0\pi & u_{Bi} + \delta_i \end{pmatrix},$$

$$V = \begin{pmatrix} -v_4\pi^\dagger & -v_3\pi \\ t_1 & -v_4\pi^\dagger \end{pmatrix},$$

$$W = \begin{pmatrix} 0 & t_2 \\ 0 & 0 \end{pmatrix}.$$

Here $v_i = \sqrt{3}a_G t_i/(2\hbar)$ $(i = 0,3,4)$ and the subscripts Ai, Bi repreent two sublattices in ith layer. The term $\pi = \hbar(\nu k_x + ik_y)$ is defined by the valley index $\nu = \pm 1$ using the wave vector $\vec{k} = (k_x, k_y)$ measured from Dirac points $K_\nu = \left(\frac{4\pi\nu}{3a_G}, 0\right)$. The diagonal site potentials are

$$u_{A1} = u_{B7} = 0\ eV$$
$$u_{A7} = u_{B1} = 0.0122\ eV$$
$$u_{Ai} = u_{Bi} = -0.0164\ eV\ (1 < i < 7),$$

And $\delta_i = (4 - i)\Delta$ (for $1 \leq i \leq 7$) introduces the interlayer potential difference (Δ) between contiguous layers through a perpendicular external electric field. The effective tight-binding parameters are $t_0 = 3.1$ eV, $t_1 = 0.3561$ eV, $t_2 = -0.0083$ eV, $t_3 = 0.293$ eV, $t_4 = 0.144$ eV, which represent hopping terms between different sites.

The character of moiré superlattice is captured by adding V_{mo} acting on both (or one of) the top and bottom layers of rhombohedral 7L graphene[18]. The reciprocal lattice vectors of graphene ($\vec{g}^G$), h-BN ($\vec{g}^{BN}$) and moiré superlattice ($\vec{G}$) are respectively given by

$$\vec{g}_m^G = \hat{R}_{\frac{\pi(m-1)}{3}} \left(0, \frac{4\pi}{\sqrt{3}a_G}\right)^\mathrm{T}$$

$$\vec{g}_m^{BN} = \hat{R}_{\frac{\pi(m-1)}{3}} \left(0, \frac{4\pi}{\sqrt{3}a_{BN}}\right)^\mathrm{T}$$

$$\vec{G}_m = \hat{R}_\theta \vec{g}_m^{BN} - \vec{g}_m^G, \qquad m \in \{1,2,\dots,6\},$$

where $a_{BN} = 0.250$ nm is the lattice constant of h-BN. The θ represents the relative twist angle between the graphene and h-BN layers. $\hat{R}_\varphi = \begin{pmatrix} \cos\varphi & -\sin\varphi \\ \sin\varphi & \cos\varphi \end{pmatrix}$ rotates a vector by angle φ. We use $\xi = \pm 1$ to distinguish the two possible alignments between graphene and h-BN. It represents the perturbation of the low-energy A (bottom) or B (top) sites in graphene by h-BN in two different ways,

giving rise to different band structures[17].

Then, we can express the V_{mo} operator as a matrix in ***k***-space with basis (*A1, B1*) or (*A7, B7*),

$$< \vec{k} + \xi\vec{G}_m \, |V_{mo}| \vec{k} > \\ = V_{AA}^{\xi}\left(\frac{I+\xi\sigma_z}{2}\right) + V_{BB}^{\xi}\left(\frac{I-\xi\sigma_z}{2}\right) \\ + \frac{\left[V_{BA}^{\xi}\delta_{v,1} + V_{AB}^{\xi}\delta_{v,-1}\right](\sigma_x, \xi\sigma_y)M\vec{G}_m}{|\vec{G}_1|}$$ ... Supplementary Equation 2

where $\sigma_{x,y,z}$ are the Pauli matrices,

$$V_{AA}^{\xi} = C_{AA}\, e^{(-1)^{m+1}\xi\phi_{AA}i}$$
$$V_{BB}^{\xi} = C_{BB}\, e^{(-1)^{m+1}\xi\phi_{BB}i}$$

$$V_{BA}^{\xi} = \left(V_{AB}^{\xi}\right)^{*} = -\xi C_{AB} e^{(-1)^{m+1}\xi\left(\phi_{AB}-\frac{\pi}{6}\right)i}$$

$$M = \frac{1}{\sqrt{\alpha^2 - 2\alpha\cos\theta + 1}}\begin{pmatrix} -\alpha\sin\theta & -\alpha\cos\theta + 1 \\ \alpha\cos\theta - 1 & -\alpha\sin\theta \end{pmatrix}$$

with $C_{AA} = -14.88$ meV, $C_{BB} = 12.09$ meV, $C_{AB} = 11.34$ meV, $\phi_{AA} = 50.19°$, $\phi_{BB} = -46.64°$, $\phi_{AB} = 19.6°$, $\alpha = a_G/a_{BN}$.

The band calculation of the intrinsic rhombohedral 7L graphene is very similar to that of bilayer graphene. As for the moiré superlattice, the original band are reconstructed into a small moiré Brillouin zone (MBZ), which is a hexagon with its center ($\tilde{\Gamma}$ point) and one corner ($\tilde{K}$ point) located at adjacent corners of the BZs of graphene and h-BN, respectively. To calculate the band structure in the MBZ, for each momentum $\vec{k}$ in it, we build a large matrix $H(\vec{k})$ whose bases include states for $3n(n+1)+1$ momentum points $\vec{k}'$ which satisfy

$$\vec{k}' = \vec{k} + \sum_{m=1}^{6} c_m\vec{G}_m, \qquad c_m \in \mathbb{N}, \ \sum_{m=1}^{6} c_m \le n.$$

Here n is the truncation length. The diagonal blocks in $H(\vec{k})$ are copies of Hamiltonian in Supplementary Equation 1 with different momentum $\vec{k}'$. And the non-diagonal blocks is where the intralayer moiré potential V_{mo} performs, following Supplementary Equation 2. Selecting $n = 2$ and diagonalizing $H(\vec{k})$ give the precise enough energy dispersion.

Supplementary Figures

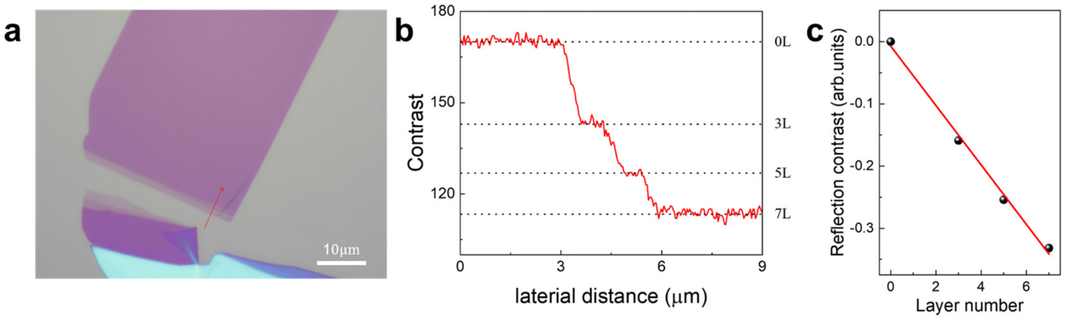

Supplementary Figure 1 | Identification of layer number of multilayer graphene. **a**, Optical image of a typical 7L graphene. **b**, Cross-sectional profile of optical contrast along the red line in (**a**). The step-like edge helps us identify the layer number accurately. **c**, layer-dependent reflection contrast. The red line is a fit to the date using the Beer-Lambert law. The layer-number-dependent optical contrast facilitates the determination of layer number quickly and reliably, compared with other techniques such as Raman spectroscopy.

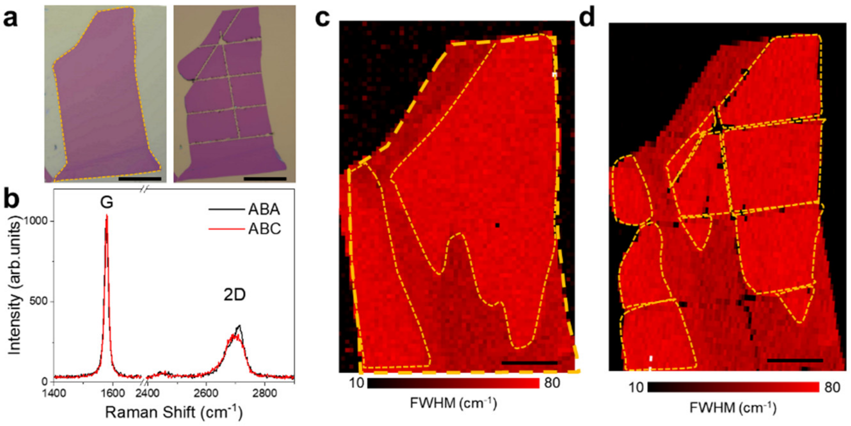

Supplementary Figure 2 | Raman characterization before and after cutting process. **a**, Optical images of multilayer graphene before and after cut by a tungsten tip. **b**, Comparison of Raman spectra acquired in ABA and ABC domains. The stacking order can be identified through the shape of 2D peak and the position of G peak. **c**, **d**, Raman mapping of the full width at half maximum (FWHM) of 2D peak for the flake before (**c**) and after (**d**) cut. The cutting process does not significantly change the domain distribution. Therefore, it can be used to stabilize ABC domain during the transfer process. The scale bars in (**a**) are both 20 μm. The scale bars in (**c**) and (**d**) are 10 μm.

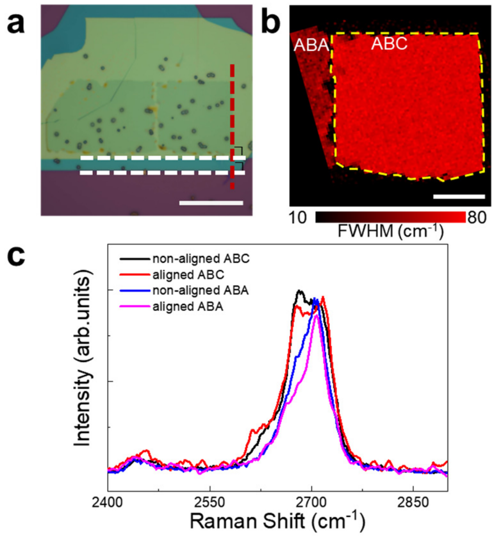

Supplementary Figure 3 | Raman characterization of h-BN encapsulated rhombohedral 7L graphene. **a**, Optical image of the 7L graphene encapsulated by h-BN. The dashed lines mark the alignment between graphene, top and bottom h-BN. The straight edge of graphene is perpendicular to both top and bottom h-BN, indicating the stack is doubly aligned. This stack is the one for device D2 in the main text. The scale bar is 25 μm. **b**, Raman map of the full width half maximum (FWHM) of 2D band peak for the sample in (**a**). The scale bar is 10 μm. **c**, Normalized Raman spectra around 2D band peak for aligned and non-aligned ABA- and ABC-stacked 7L graphene. All the samples are encapsulated by h-BN.

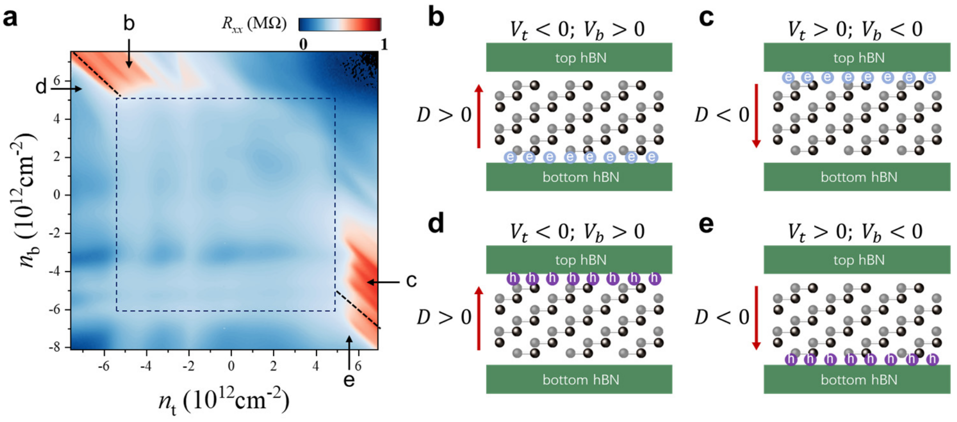

Supplementary Figure 4 | Surface states and screening effect in aligned rhombohedral 7L graphene. **a**, Longitudinal resistance R_{xx} as a function of carrier density n_t and n_b induced by top and bottom gate, respectively, measured at $T = 50$ mK and $B = 0$ T. The data are the same as that in Fig. 2b, but plotted as $n_t = \frac{C_t \Delta V_t}{e}$ and $n_b = \frac{C_b \Delta V_b}{e}$. A series of horizontal and vertical lines were observed inside the region marked by the dashed box, which indicates that the two surface states are electronically decoupled and effectively screened out by each other. Under large displacement field ($|D| > |D_c|$), layer-polarized surface states dominate, namely, only one of the two surfaces contributes to the conduction and the other one is fully depleted. The four kinds of layer-polarized surface states marked by the arrows are schematically shown in **b-e**, respectively. In these states, both gates can effectively tune the individual surface state.

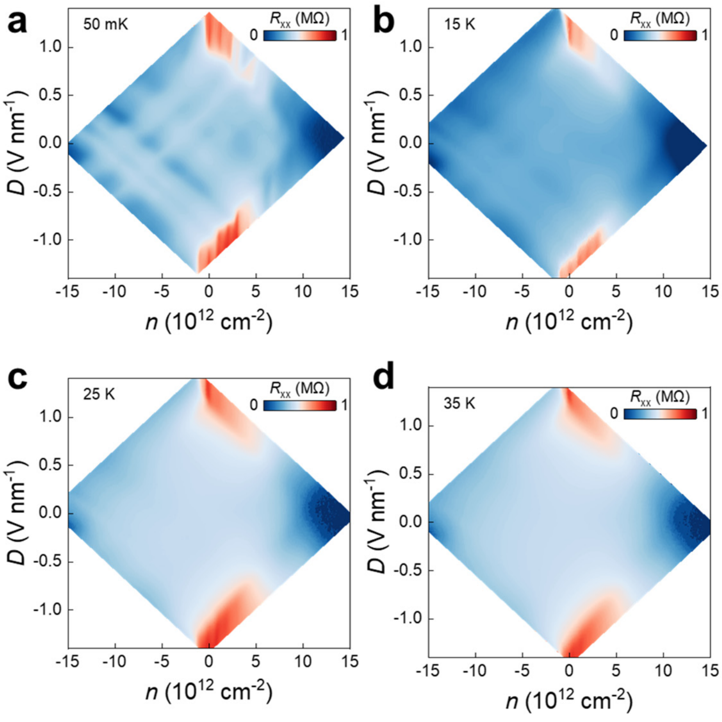

Supplementary Figure 5 | Temperature dependence of $R_{xx}(n, D)$ mapping for the aligned rhombohedral 7L graphene. Color maps of longitudinal resistance R_{xx} as a function of carrier density n and displacement field D measured at $T = 50$ mK (**a**), 15 K (**b**), 25 K (**c**), 35 K (**d**). The data were measured in Device D2 at $B = 0$ T.

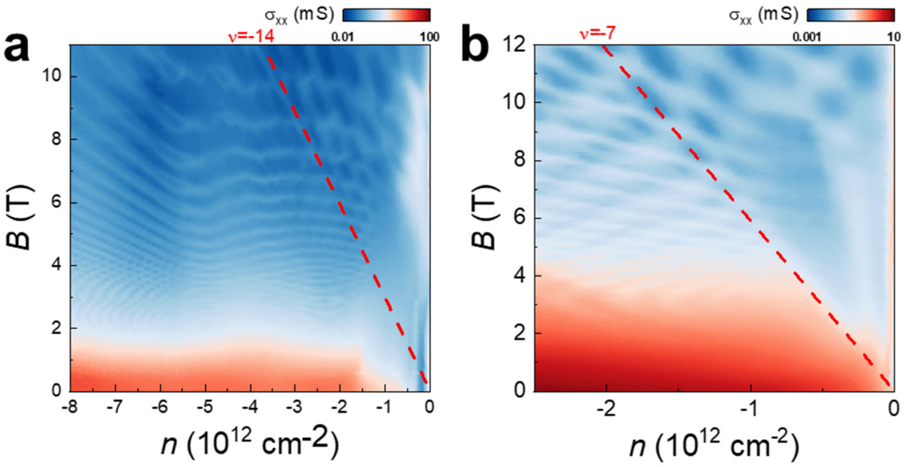

Supplementary Figure 6 | Landan fan diagrams of non-aligned rhombohedral 7L graphene. **a**, **b**, Longitudinal conductivity $\sigma_{xx}(n, B)$ mapping as a function of n and B at fixed $D = 0$ V nm^{-1} (**a**) and $D = -0.55$ V nm^{-1} (**b**). At $D = 0$ V nm^{-1}, pronounced $\nu = -14$ corresponding to the gap between zeroth LL and first LL in the valence band was observed. Under non-zero D, valley degeneracy is lifted due to the inversion symmetry breaking, leading to the appearance of pronounced $\nu = -7$. Both (**a**) and (**b**) demonstrate the existence of 7π Berry phase in intrinsic rhombohedral 7L graphene. The data were measured in Device D3.

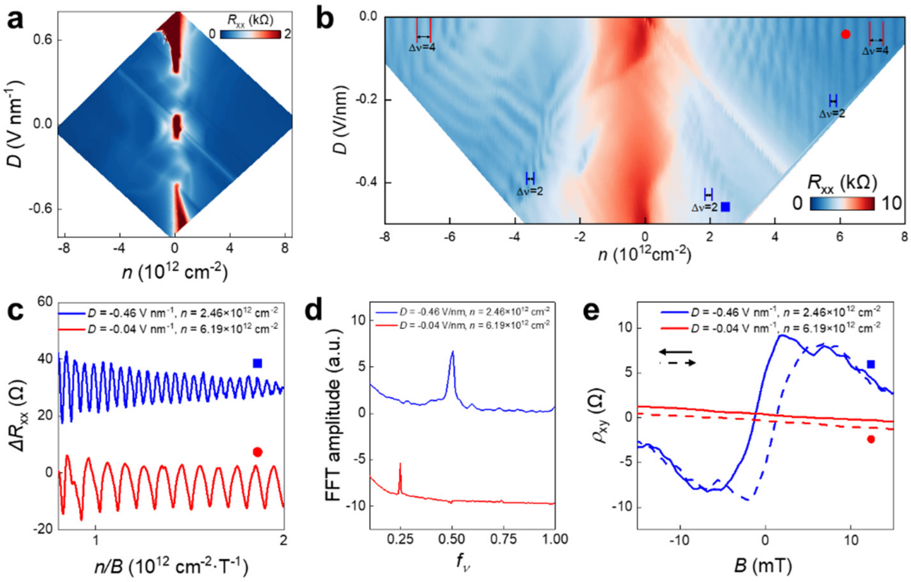

Supplementary Figure 7 | Spontaneous symmetry breaking in non-aligned rhombohedral 7L graphene. a, Color maps of longitudinal resistance R_{xx} as a function of carrier density n and displacement field D measured at $B = 0$ T for the devices without moiré superlattice (Device 3). **b**, $R_{xx}(n, D)$ mapping at $B = 4$ T. Quantum oscillations with different degeneracies were observed. At high n, typical degeneracy of 4 (2 spins $\times$ 2 valleys) in graphene is marked with red lines. At low n, half metal with degeneracy of 2 was observed, which is marked with blue lines. **c**, SdH oscillations at normal and half metal states. The data were taken at the position marked with circle (red) and square (blue) labels in (**b**). **d**, The corresponding fast Fourier transform (FFT) of the SdH oscillations in (**c**). The data are plotted as a function of $f_\nu = f_B/(\phi_0 n)$, where f_B is the oscillation frequency in the unit of tesla, $\phi_0 = h/e$ is the magnetic flux quantum. **e**, Low-field anti-symmetrized Hall resistance ρ_{xy} as a function of B measured at normal and half metal states labeled as those in (**c**) and (**d**). AHE with hysteresis loops was observed in the half metal state, indicating it is spontaneous valley polarized state. All the data were taken at $T = 50$ mK in Device D3.

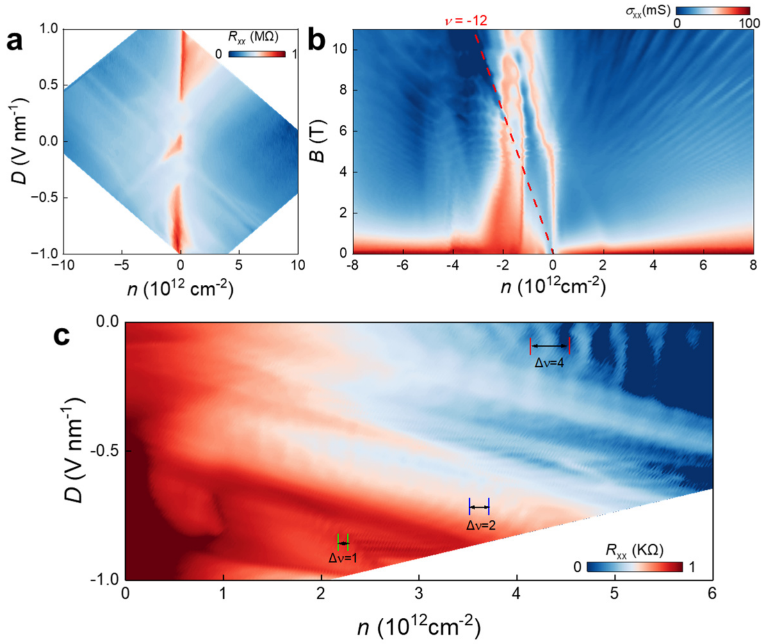

Supplementary Figure 8| Phase diagram and spontaneous symmetry breaking in non-aligned rhombohedral 6L graphene (Device D5). **a**, Color maps of longitudinal resistance R_{xx} as a function of n and D measured at $B = 0$ T. **b**, Longitudinal conductivity $\sigma_{xx}(n, B)$ mapping as a function of n and B at fixed $D = 0$ V nm^{-1}. We observed pronounced $\nu = -12$ corresponding to the gap between zeroth LL and first LL in the valence band, consistent with the layer number of 6. **c**, $R_{xx}(n, D)$ mapping at $B = 4$ T. Quantum oscillations with degeneracies of 4, 2, 1 were observed and marked. The lift of degeneracy reveals that spontaneous symmetry breaking occurs at half-metal ($\Delta\nu = 2$) and quarter-metal states ($\Delta\nu = 1$). All the data were taken at $T = 1.5$ K in Device D5.

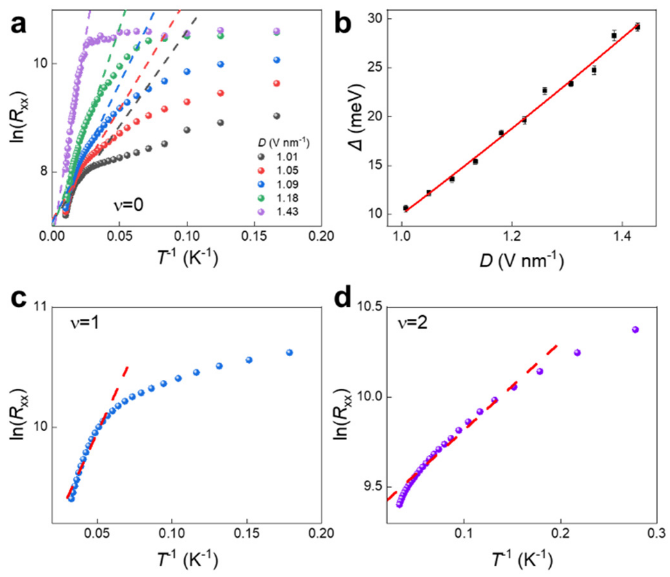

Supplementary Figure 9 | Arrhenius plot and extracted gap size of aligned rhombohedral 7L graphene. **a**, $\ln(R_{xx})$ as a function of T^{-1} under various displacement field at charge-neutrality points ($\nu = 0$). The dashed lines are the linear fits, which can be used to extract transport gap

according to thermal activation equation $1/R_{xx} \propto e^{-\Delta/2k_BT}$. **b**, The measured gap size as a function of D at charge-neutrality points. **c**, **d**, $\ln(R_{xx})$ as a function of T^{-1} at quarter filling ($\nu = 1$) and half filling ($\nu = 2$) under $D = 1.1$ V nm^{-1}. Linear fittings are obtained at relative high temperature regions. Since the correlated gap at $\nu = 2$ has quite small size, the linear fit is not very accurate. To precisely measure the gap at $\nu = 2$, much higher D should be applied to enhance the gap size. These analyses are based on the data in Device D2.

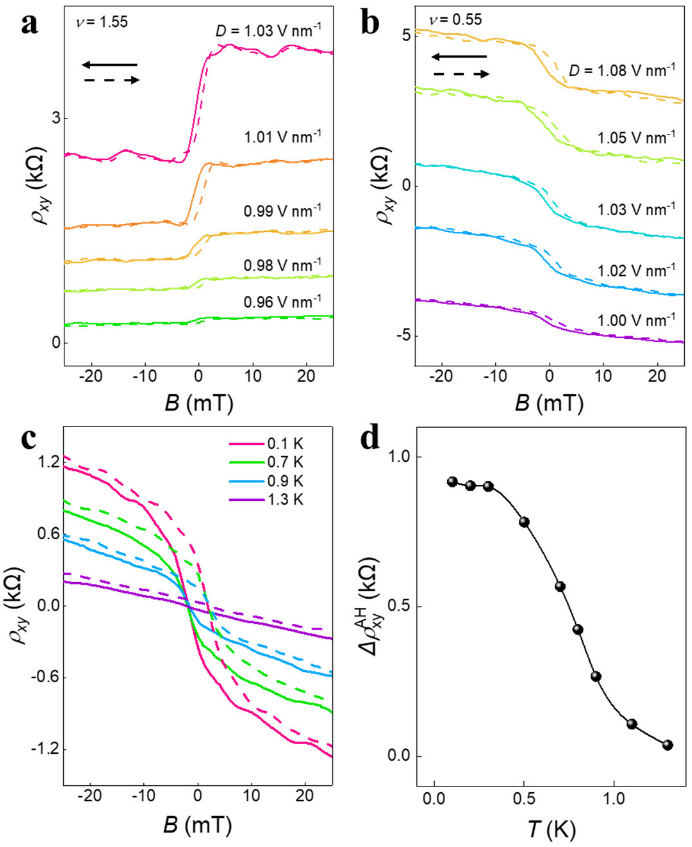

Supplementary Figure 10 | Additional data of AHE with hysteresis loops. **a**, **b**, Anti-symmetrized Hall resistance ρ_{xy} as a function of B swept back and forth at low field regions at (**a**) a fixed $\nu = 1.55$, varying D from 1.03 V nm^{-1} to 0.96 V nm^{-1} and (**b**) a fixed $\nu = 0.55$, varying D from 1.08 V nm^{-1} to 1.00 V nm^{-1}. The absolute values are manually offset for clarity. **c**, Temperature dependence of AHE. ρ_{xy} as a function of B swept back and forth, showing ferromagnetic hysteresis at different temperatures, with fixed $\nu = 0.54$ and $D = 1.03$ V nm^{-1}. **d,** The corresponding residual Hall resistance $\Delta\rho_{xy}^{AH}$ as a function of temperature.

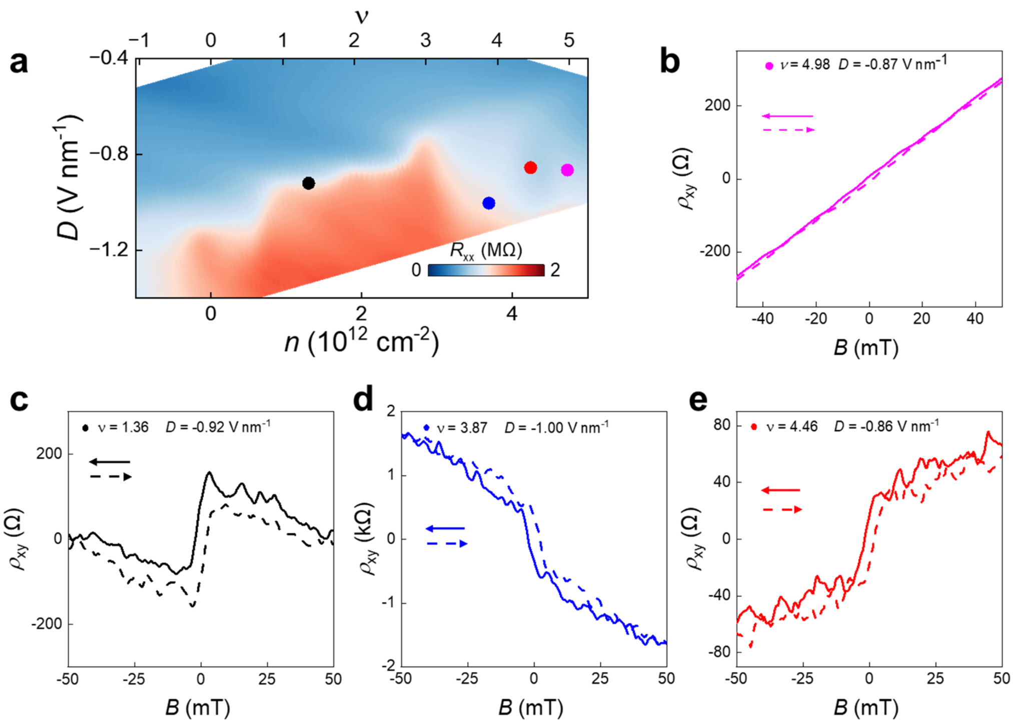

Supplementary Figure 11 | Ferromagnetic states at negative *D*. **a**, Fine $R_{xx}(n, D)$ mapping at negative D side. **b**, Normal state far away from moiré flat band, showing the linear Hall effect without hysteresis loop. **c-e**, Anti-symmetrized Hall resistance ρ_{xy} as a function of B swept back and forth at three position marked with different colors in (**a**): $\nu = 1.36$, $D = -0.92$ V nm^{-1} (**c**); $\nu = 3.87$, $D = -1.00$ V nm^{-1} (**d**); $\nu = 4.46$, $D = -0.86$ V nm^{-1} (**e**). AHE with both nonlinear features and hysteresis loops was observed. The data were taken in Device D2.

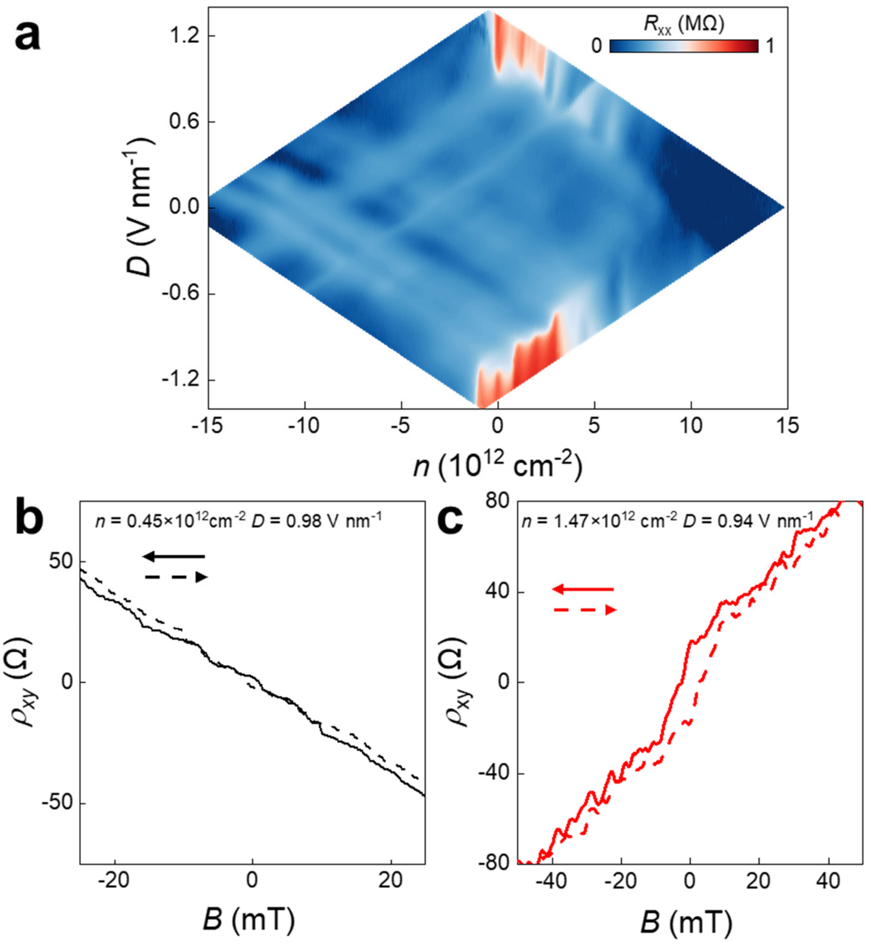

Supplementary Figure 12 | Layer-polarized ferromagnetic states at a second aligned device (Device D4). a, Color maps of longitudinal resistance R_{xx} as a function of carrier density n and displacement field D measured at $T = 50$ mK and $B = 0$ T. This device shows similar moiré period to Device 2 with almost identical features as those in Fig. 2b, indicating the high homogeneity and reproducibility in graphene/h-BN moiré superlattice. **b**, Normal state showing the linear Hall effect without hysteresis loop as a comparison. **c**, Anti-symmetrized Hall resistance ρ_{xy} as a function of B swept back and forth at $n = 1.47 \times 10^{12}$ cm^{-2} and $D = 0.94$ V nm^{-1}. AHE with both nonlinear features and hysteresis loops was observed at polarized surface states (high D and nonzero n), consistent with that in Device D2.

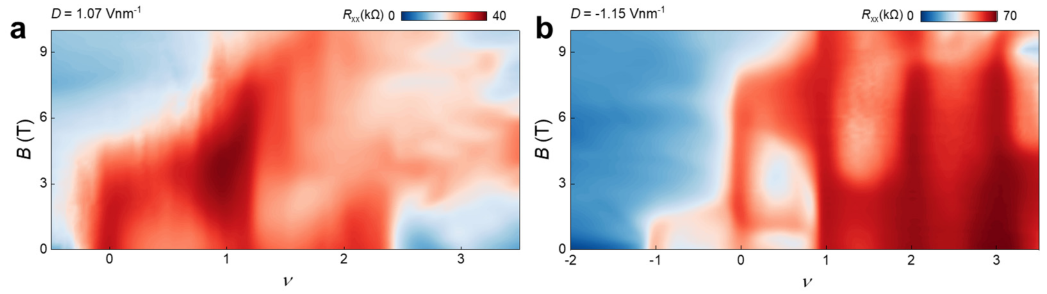

Supplementary Figure 13 | Landau fan diagrams under large displacement fields. a, At fixed $D = 1.07$ V nm^{-1}. **b**, At fixed $D = -1.15$ V nm^{-1}. The failure of the observations of quantum oscillations is due to the relatively low carrier mobility inside the flat band. We observed all the

insulating peaks are independent on magnetic fields in terms of position, i.e., $\frac{\partial n}{\partial B} = 0$. According to the Streda formula $\frac{\partial n}{\partial B} = C\frac{e}{h}$, we can get the Chern numbers of the moiré bands are $C = 0$. The data were taken in Device D2.

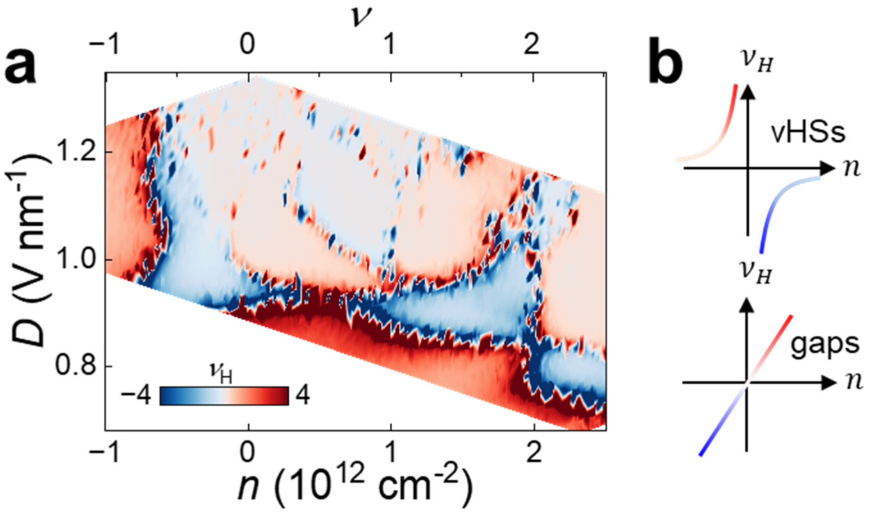

Supplementary Figure 14| a, Normalized Hall density ν_{H} as a function of *n* and *D* at $B = \pm 1$ T. The ν_H is defined as $\nu_H = 4n_H/n_s$, where $n_H = -[e(dR_{xy}/dB)]^{-1}$and n_s is the carrier density at full filling. The plot is obtained by converting R_{xy} to ν_H in Fig. 4b using above definition. **b**, Schematic of sign reversal of ν_H near gaps and vHSs.

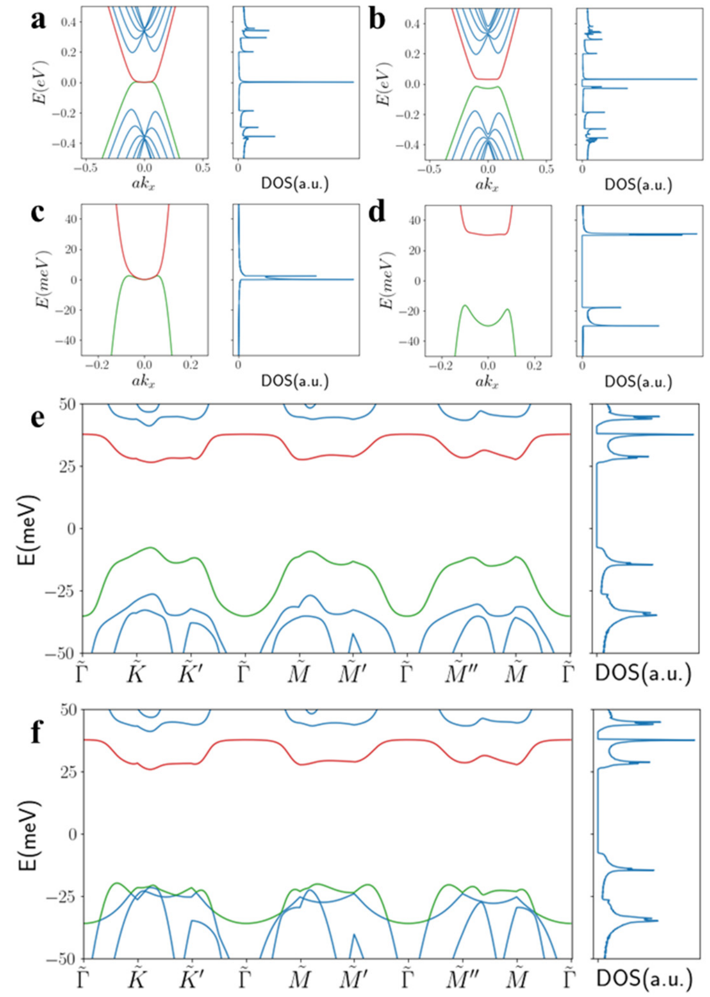

Supplementary Figure 15 | Band structure and DOS of rhombohedral 7L graphene. a-d, Calculated band structure and DOS of intrinsic rhombohedral 7L graphene without moiré superlattice. (**a**) and (**c**) are band structures without interlayer potential. (**b**) and (**d**) are band structures under interlayer potential $\Delta = 10$ mV. (**c**) and (**d**) are the low-energy surface bands. **e**, Calculated band structure of rhombohedral 7L graphene with moiré potential at both top and bottom surface. **f**, Calculated band structure of rhombohedral 7L graphene with moiré potential at only one of the two surfaces. The similar low energy conduction band in (**e**) and (**f**) indicates that the two surfaces are almost decoupled. Right panels in (**a**)-(**f**) are the corresponding DOS as a function of E.

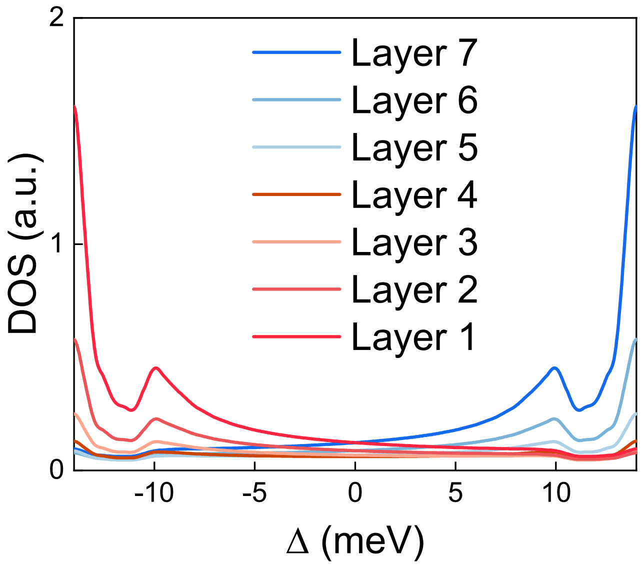

Supplementary Figure 16 | Layer-dependent DOS distribution. The plot shows DOS distribution in each layer as a function of interlayer potentials for doubly aligned rhombohedral 7L graphene. The Fermi energy is fixed at 50 meV to include all the DOS contributions from the first conduction moiré band. Layer 1 and Layer 7 represent the bottom layer and top layer, respectively.

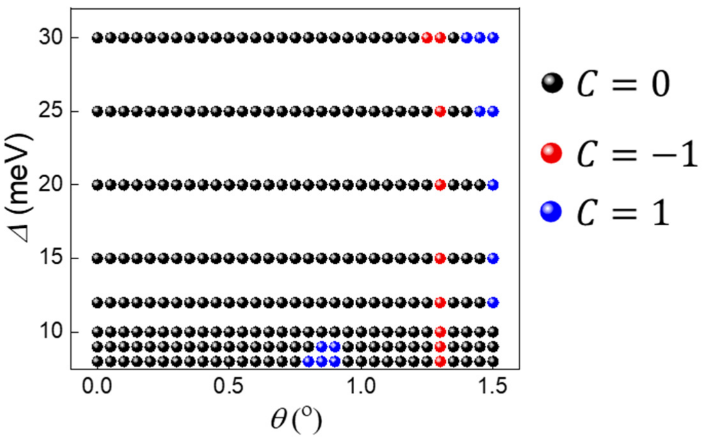

Supplementary Figure 17 | Calculated Chern number of the first moiré conduction band in doubly aligned rhombohedral 7L graphene. Only interlayer potentials Δ larger than 8 meV are shown, since at low Δ the moiré flat bands are not fully isolated. The results show zero Chern number of the band at large region of twist angles and Δ. Our observations of ferromagnetic states are located at zero Chern number region.

Supplementary References

1 Ni, Z. H. *et al.* Graphene thickness determination using reflection and contrast spectroscopy. *Nano Lett.* **7**, 2758-2763 (2007).

2 Eckmann, A. *et al.* Raman fingerprint of aligned graphene/h-BN superlattices. *Nano Lett.* **13**, 5242-5246 (2013).

3 Cheng, B. *et al.* Raman spectroscopy measurement of bilayer graphene's twist angle to boron nitride. *Appl. Phys. Lett.* **107**, 033101 (2015).

4 Finney, N. R. *et al.* Tunable crystal symmetry in graphene-boron nitride heterostructures with coexisting moire superlattices. *Nat. Nanotechnol.* **14**, 1029-1034 (2019).

5 Mullan, C. *et al.* Mixing of moiré-surface and bulk states in graphite. *Nature* **620**, 756-761 (2023).

6 Dean, C. R. *et al.* Hofstadter's butterfly and the fractal quantum Hall effect in moiré superlattices. *Nature* **497**, 598-602 (2013).

7 Cao, Y. *et al.* Correlated insulator behaviour at half-filling in magic-angle graphene superlattices. *Nature* **556**, 80-84 (2018).

8 Krishna Kumar, R. *et al.* High-temperature quantum oscillations caused by recurring Bloch states in graphene superlattices. *Science* **357**, 181-184 (2017).

9 Wang, Z. *et al.* Composite super-moiré lattices in double-aligned graphene heterostructures. *Sci. Adv.* **5**, eaay8897 (2019).

10 Yang, Y. *et al.* In situ manipulation of van der Waals heterostructures for twistronics. *Sci. Adv.* **6**, eabd3655 (2020).

11 Yin, J. *et al.* Dimensional reduction, quantum Hall effect and layer parity in graphite films. *Nat. Phys.* **15**, 437-442 (2019).

12 Waters, D. *et al.* Mixed-dimensional moiré systems of twisted graphitic thin films. *Nature* **620**, 750-755 (2023).

13 Shi, Y. *et al.* Electronic phase separation in multilayer rhombohedral graphite. *Nature* **584**, 210-214 (2020).

14 Zhou, H. *et al.* Half- and quarter-metals in rhombohedral trilayer graphene. *Nature* **598**, 429-433 (2021).

15 Liu, K. *et al.* Spontaneous broken-symmetry insulator and metals in tetralayer rhombohedral graphene. *Nat. Nanotechnol.* **19,** 188-195 (2024).

16 Han, T. *et al.* Correlated insulator and Chern insulators in pentalayer rhombohedral-stacked graphene. *Nat. Nanotechnol.* **19,** 181-187 (2024).

17 Chittari, B. L., Chen, G., Zhang, Y., Wang, F. & Jung, J. Gate-tunable topological flat bands in trilayer graphene boron-nitride moiré superlattices. *Phys. Rev. Lett.* **122**, 016401 (2019).

18 Jung, J., DaSilva, A. M., MacDonald, A. H. & Adam, S. Origin of band gaps in graphene on hexagonal boron nitride. *Nat. Commun.* **6**, 6308 (2015).
